# Tubular injury in diabetic kidney disease: molecular mechanisms and potential therapeutic perspectives

**DOI:** 10.3389/fendo.2023.1238927

**Published:** 2023-08-02

**Authors:** Yu Wang, Mingyue Jin, Chak Kwong Cheng, Qiang Li

**Affiliations:** ^1^ Department of Endocrinology and Metabolism, Shenzhen University General Hospital, Shenzhen, Guangdong, China; ^2^ Department of Biomedical Sciences, City University of Hong Kong, Hong Kong, Hong Kong SAR, China; ^3^ School of Biomedical Sciences, The Chinese University of Hong Kong, Hong Kong, Hong Kong SAR, China

**Keywords:** tubular injury, diabetic kidney disease, mechanism, therapy, programmed cell death, stem cell

## Abstract

Diabetic kidney disease (DKD) is a chronic complication of diabetes and the leading cause of end-stage renal disease (ESRD) worldwide. Currently, there are limited therapeutic drugs available for DKD. While previous research has primarily focused on glomerular injury, recent studies have increasingly emphasized the role of renal tubular injury in the pathogenesis of DKD. Various factors, including hyperglycemia, lipid accumulation, oxidative stress, hypoxia, RAAS, ER stress, inflammation, EMT and programmed cell death, have been shown to induce renal tubular injury and contribute to the progression of DKD. Additionally, traditional hypoglycemic drugs, anti-inflammation therapies, anti-senescence therapies, mineralocorticoid receptor antagonists, and stem cell therapies have demonstrated their potential to alleviate renal tubular injury in DKD. This review will provide insights into the latest research on the mechanisms and treatments of renal tubular injury in DKD.

## 1 Introduction

Diabetic kidney disease (DKD) is one of the microvascular complications of diabetes mellitus (DM), imposing a huge economic burden on individuals and public health systems (1). DKD affects nearly one third of DM patients, and the ten-year cumulative mortality rate is as high as 31.1% (2). However, the underlying mechanisms of DKD remain elusive. In-depth research into the pathological mechanisms of DKD should lead to the identification of new diagnostic markers and the development of therapeutic strategies. Although glomerulosclerosis is an important pathological manifestation of DKD, the rate of renal function loss is more closely correlated with tubular injury and interstitial fibrosis (3). There is substantial evidence that DKD can be classified as either albuminuric or non-albuminuric. Non-albuminuric DKD is characterized by significant tubulointerstitial damage and fibrosis without overt glomerulopathy. In addition, overt proteinuria is absent in a significant proportion of patients during the progression of DKD to ESRD, highlighting the distinct pathological roles of tubular cell damage and glomerular damage (4). Diabetic patients experience a variety of structural abnormalities in their renal tubules, including renal tubular atrophy, interstitial fibrosis and peritubular capillary rarefaction, all of which are directly related to the decline in renal function (5). Renal proximal tubular epithelial cells (PTECs) dysfunction is increasingly implicated in the pathogenesis and progression of DKD. Moreover, some previous studies have shown that proximal tubular injury precedes microalbuminuria and the onset of early glomerulopathy in early DKD (6). In addition, the accumulation of albuminuria induces oxidative stress and inflammatory responses in tubular epithelial cells (TECs), leading to morphological and functional changes, epithelial-mesenchymal transition and apoptosis in TECs. These adverse events ultimately promote renal fibrosis and ESRD (7). This review aims to provide an updated overview of the pathophysiology and therapeutic strategies against renal tubular dysfunction in DKD.

## 2 Renal tubular changes in DKD

**Figure 1:**
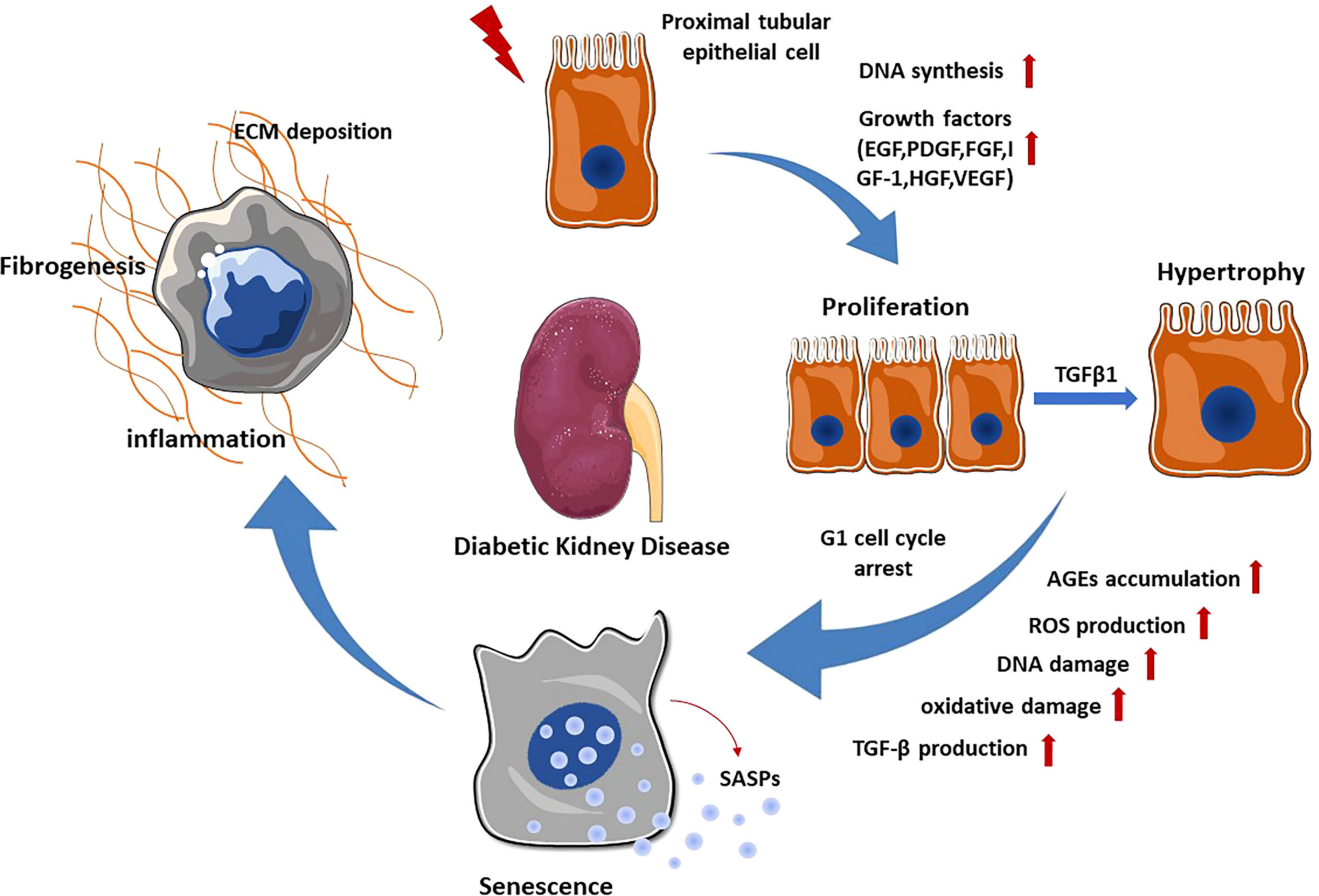
Renal tubular epithelial cell changes in DKD. When exposed to various stimuli, PTECs express and secrete multiple growth factors, which promote the proliferation of PTECs. Subsequently, TGF-β1 stimulates the transformation of PTECs from proliferation to hypertrophy through ERK and P38 pathway. In order to prevent excessive proliferation, PTECs undergo G1 cell cycle arrest and transition to senescence. Senescent PTECs can secrete SASPs, promoting renal inflammation and fibrosis.

Almost 90% of the renal parenchyma consists of renal tubules and tubulointerstitial tissue. Due to their location and major resorptive role within the nephron, PTECs are exposed to various factors in the glomerular filtrate, peritubular capillary blood supply and tubulointerstitium. They can therefore be injured by a variety of pro-inflammatory and pro-fibrotic substances that cause tubular injury. In particular, the major histological changes in early DKD include tubular cell hypertrophy, thickening of the tubular basement membrane and interstitial inflammation with mononuclear cell infiltration. Progression of these early tubular abnormalities leads to interstitial fibrosis and tubular atrophy. In the STZ-induced diabetic mouse model, DNA synthesis increases and peaks at day 2 in the proximal tubules, and the expression of various growth factors including epidermal growth factor (EGF), platelet-derived growth factor (PDGF), basic fibroblast growth factor (FGF), insulin-like growth factor-1 (IGF-1), hepatocyte growth factor (HGF) and vascular endothelial growth factor (VEGF) has been observed (8).Together, they lead to proliferation of renal tubular cells during the early stage of DKD. Lineage tracing analysis of terminally differentiated proximal tubular epithelia revealed active cell proliferation in the kidneys of diabetic mice (9). PKCβ1 expressed in the proximal tubule is critical for inducing the expression of transforming growth factor-β1 (TGFβ1), which is an essential mediator of the switch from proliferation to hypertrophy in renal proximal tubular cells during the early stage of DKD through the extracellular signal-regulated kinase (ERK) and p38 pathway. In order to prevent excessive proliferation induced by hyperglycemia, PTECs G1 cell cycle arrest and transition to senescence in the following process (10). Cellular senescence is thought to be primarily caused by DNA damage. Under diabetic conditions, hyperglycemia mediated reactive oxygen species (ROS) production and advanced glycation end products (AGEs) accumulation cause DNA damage, which subsequently promotes senescence of renal TECs and glomerular cells (11). Senescent renal TECs could secrete senescence-associated secretory phenotypes (SASPs) such as chemokines, cytokines, growth factors and extracellular matrix proteins, leading to chronic inflammation and fibrosis (12). Notably, senescent renal TECs may induce fibroblast activation and proliferation by releasing Sonic hedgehog protein, contributing to the progression of DKD (13). These tubulointerstitial abnormalities exacerbate the development of DKD by promoting oxidative stress, inflammation, hypoxia and tubulointerstitial fibrosis (**Figure 1**).

## 3 Mechanism

**Figure 2:**
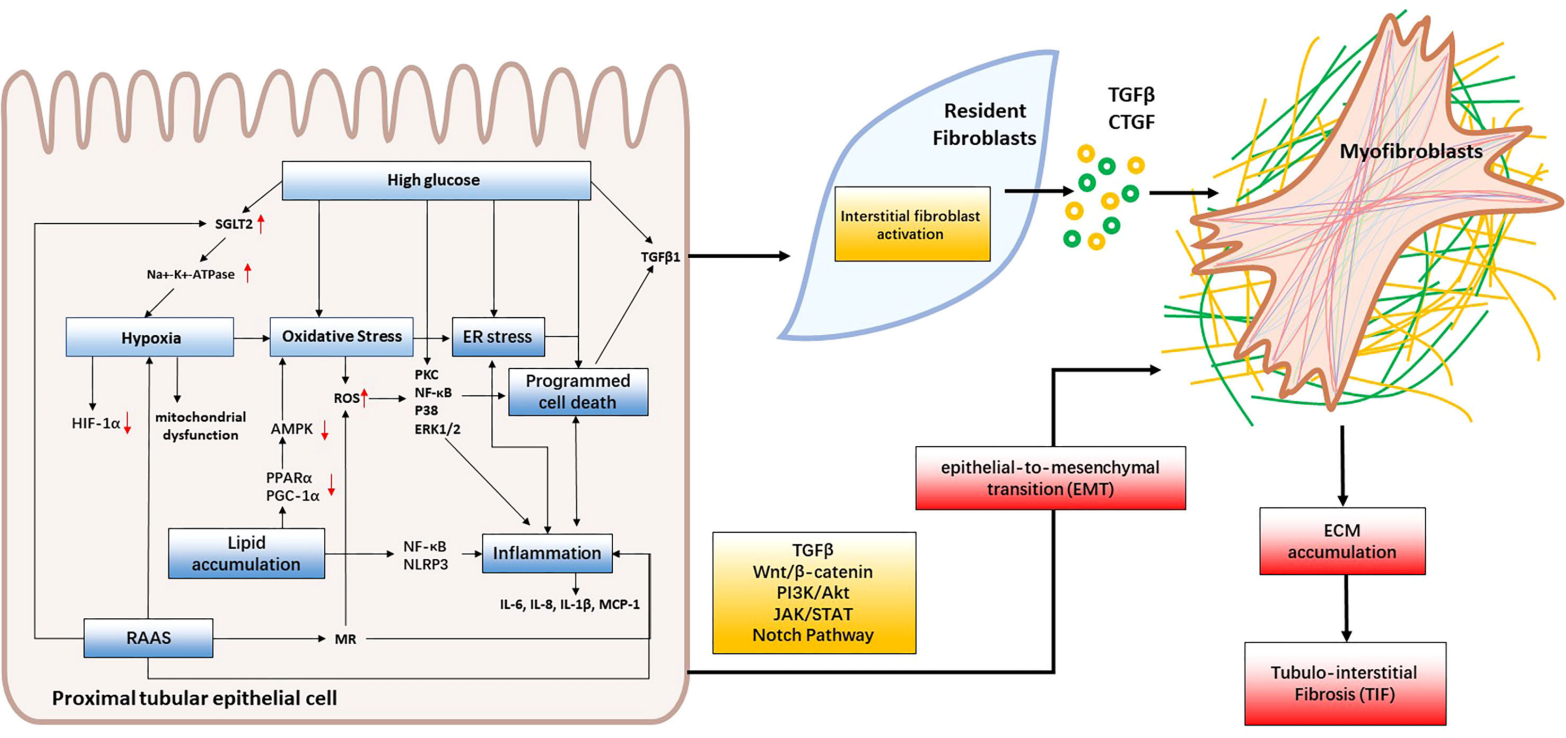
Mechanisms of renal tubular injury in DKD. Hyperglycemia, lipid accumulation and RAAS activation lead to oxidative stress, hypoxia, and ER stress, consequently triggering inflammation, programmed cell death and EMT. These cascading effects contribute to renal tubular cell injury and interstitial fibroblast activation. When intrinsic renal cells become activated, they secrete cytokines like TGF-β and CTGF that facilitate the formation of myofibroblasts which contribute to fibrosis by producing collagen I, collagen III, collagen IV, fibronectin, and laminin. This leads to the accumulation of extracellular matrix (ECM) and, ultimately, the development of tubulointerstitial fibrosis and DKD.

Although the underlying pathogenesis of diabetic kidney disease (DKD) is not fully understood, several factors have been proven to contribute to the development of tubular injury. These factors include hyperglycemia, lipid accumulation, oxidative stress, hypoxia, RAAS, ER stress, inflammation, EMT and programmed cell death (**Figure 2**). In this review, we will provide an overview of the latest studies investigating the mechanisms underlying renal tubular injury in DKD.

### 3.1 Hyperglycemia and oxidative stress

Hyperglycemia is one of the main causes of DKD and also the primary inducer of renal tubular injury. PTECs are highly sensitive to hyperglycemia (14). Chronic hyperglycemia and insulin resistance lead to a series of metabolic changes that cause the accumulation of AGEs, activation of protein kinase C (PKC)-α, -β, and -δ (15), and oxidative stress. These changes can damage the renal tubules by disrupting their normal cellular functions, such as ion transport and energy metabolism (16).

Hyperglycemia increases the amount of glucose filtered through the glomeruli, thereby increasing tubular glucose load, exposure and reabsorption. At normal blood glucose levels, up to 97% of glucose filtered by the glomeruli is reabsorbed by sodium-dependent glucose transporter-2 (SGLT2) in the initial segment of the tubule, and only 3% of the remaining glucose is reabsorbed by sodium-dependent glucose transporter-1 (SGLT1) in the later segment of the proximal tubule (17). The glucose reabsorbed by the proximal tubule cells diffuses further across the basolateral membrane and is released into the blood by glucose transporter 1 (GLUT1) and glucose transporter 2 (GLUT2) located in the S3 and S1 segments of the proximal tubule. The ability of SGLT1 and SGLT2 to transport glucose depends on their protein expression in the cell membrane. The expression of SGLT1 and SGLT2 mRNA is significantly upregulated in rat models of diabetes (18). Similarly, renal TECs freshly isolated from the urine of type 2 diabetic patients show increased renal glucose absorption along with increased mRNA and protein levels of SGLT2 and GLUT2 (19). An increased number of SGLT2+ TECs has been observed in the kidney of diabetic mice as an adaptive response to hyperglycemia. Such a response increases urinary glucose reabsorption, Na+-K+-ATPase activity and glucose oxidation, leading to hypoxia and tubular injury (20). The accumulation of matrix protein in diabetic kidneys is largely due to epithelial-to-mesenchymal transition (EMT) of tubular cells. There are ample evidences that hyperglycemia causes oxidative stress by increasing the production of ROS, which have been shown to facilitate high glucose-induced EMT in renal tubular cells (21). High glucose could promote the production of TGF-β1 and extracellular matrix (ECM) components such as fibronectin and collagen I, III, IV in renal tubular cells, thereby promoting tubulointerstitial fibrosis (22). High glucose could also induce programmed cell death such as pyroptosis, apoptosis, autophagy and ferroptosis in tubular cells, which will be described in the following part.

In summary, hyperglycemia induces renal tubules metabolic disturbances, hypoxia, oxidative stress, EMT, and programmed cell death which facilitate renal tubular injury and renal fibrogenesis.

### 3.2 Lipid accumulation

Lipid accumulation is a common feature in the tubular cells of patients with DKD (23). Oil red O staining showed that lipid accumulation was observed in the kidneys of both diabetic mouse models and DKD patients, and was mainly located in the proximal tubules (24). Lipid accumulation is likely caused by increased lipid uptake and synthesis, decreased β-oxidation and cholesterol efflux (25). During DKD, downregulation of energy metabolism genes such as Peroxisome Proliferator-Activated Receptor α and peroxisome proliferator-activated receptor gamma coactivator 1 α (PGC-1α) leads to suppressed AMP kinase (AMPK) signaling, followed by downregulation of fatty acid oxidation genes and activation of triglyceride synthesis-related genes in proximal tubular cells (26).

Lipotoxicity in renal TECs is associated with inflammation, oxidative stress, mitochondrial dysfunction and cell death (27). Fatty acid overload in mitochondria causes incomplete β-oxidation, which increases oxidative stress, induces mitochondrial dysfunction, and promotes ROS production. ROS can further drive tubular epithelial cell apoptosis and affect normal renal function (28). Reduced fatty acid oxidation and mitochondrial dysfunction lead to an increase in the AMP/ATP ratio, which has an immediate effect on renal PTECs by consuming abundant ATP (29). In addition, fatty acids can promote the activation of transcription factors nuclear factor-κB (NF-κB) and NOD-like receptor protein 3 (NLRP3) inflammatory pathways in TECs, increase the release of inflammatory factors, such as Interleukin-1β (IL-1β), Interleukin-6 (IL-6) and tumor necrosis factor-α (TNF-α), and aggravate renal tubular damage and inflammatory response (24). Phosphofurin acidic cluster sorting protein 2 (PACS-2) is a versatile sorting protein involved in the regulation of lipid metabolism, apoptosis and autophagy (30). PACS-2 is mainly expressed in renal tubules and its level was found to be decreased in kidneys of STZ-induced diabetic mice and patients with DKD (31). Tubule-specific PACS-2 deletion induces kidney injury via inhibition of cholesterol efflux and promotion of lipid synthesis (32). Kidney injury molecule 1 (KIM-1) is an immunoglobulin superfamily protein that is significantly upregulated in the injured proximal tubule, and the expression of KIM-1 is increased in the kidney, blood and urine of patients with DKD (33). It has been reported that KIM-1 mediates fatty acid uptake by proximal tubular cells and induces cell death, mitochondrial fission and the DNA damage response in these cells (34).

Hypercholesterolemia also results in lipid deposition in kidney tissue, further compromising oxygen delivery through diffusion (35). Additionally, lipid deposition in renal arteries increases their stiffness and reduces their ability to dilate and deliver oxygenated blood when oxygen tensions are low (36). In conclusion, lipid accumulation plays multiple roles in the pathogenesis of renal tubular injury of DKD, including impairing tubular cell function, increasing oxidative stress and mitochondrial dysfunction, inducing inflammation, promoting tubular cell death and exacerbating hypoxia.

### 3.3 Hypoxia

Renal hypoxia has been observed in animal models of diabetes (37). In DKD, a vicious circle of insufficient oxygen supply, increased oxygen consumption and abnormalities in oxygen utilization is created. As a microvascular complication of diabetes, the characteristic lesions of DKD include thickening of the basement membrane of renal capillaries and vascular damage, resulting in reduced blood flow and oxygen delivery to the kidneys. In addition, increased levels of angiotensin II (Ang II) and endothelin, as well as a deficiency in nitric oxide production, may further induce vasoconstriction and ischemia, which are responsible for hypoxia (38).

The enormous oxygen consumption in the kidneys is largely due to tubular reabsorption. Renal TECs are vulnerable to the diabetic state due to their high energy requirements and dependence on aerobic metabolism. In the early stages of DKD, increased hyper-reabsorption and gluconeogenesis expose proximal tubular cells to hypoxia, resulting in mitochondrial dysfunction, increased oxidative stress and tubular injury (39). Hypoxia in renal tubular cells can cause tubular cell expansion, degeneration, apoptosis and cytokine production and secretion, thereby activating interstitial fibroblasts, increasing extracellular matrix production and promoting the progression of tubulointerstitial fibrosis (40). Hypoxia has a significant effect on the regulation of a wide variety of molecules, including growth factors, hormones, vasoactive compounds, and enzymes involved in intermediary metabolism (41). Hypoxia-inducible factor 1α (HIF-1α) is a key mediator that allows cells to adapt to hypoxia by increasing angiogenesis, improving oxygen delivery and modulating aspects of cellular metabolism. HIF-1α is mainly expressed in tubular and glomerular epithelial cells in response to hypoxia (42). It has been postulated that HIF-1α modulates fibrogenesis-related genes. Hypoxia-induced dysregulated expression of HIF-1α contributes to the development of interstitial fibrosis. Interstitial fibrosis further induces ischemia and hypoxia by impairing capillary to tubule diffusion, creating a vicious cycle and exacerbating renal injury (43).

Mitochondria is the primary organelle of in PTECs that is modulated to oxidative stress and hypoxic injury in the diabetic state (44). Time-series proteomic study was performed by exposing human PTECs to hypoxia/high glucose conditions. The study result has shown that the expression of various enzymes involved in oxidative phosphorylation and ATP production (SLC25A5, ATP5B, ATP5A1) was decreased with time, indicating a gradual mitochondrial dysfunction that could contribute to the development of DKD (45).

Although there are a large number of *in vitro* and *in vivo* studies investigating the mechanism of hypoxia-induced renal tubular damage, due to the lack of non-invasive means, there are few clinical studies that have evaluated renal hypoxia in detail. With the development of blood oxygen level-dependent MRI technology, it is expected to further elucidate the pathophysiological mechanism of hypoxia-induced renal tubular injury in DKD. In conclusion, hypoxia is an important factor leading to tubular injury in DKD. It may cause damage and apoptosis of TECs through multiple mechanisms, including mitochondrial dysfunction, oxidative stress, regulation of HIF-1α, and alteration of extracellular matrix (46). One strategy to prevent and treat DKD is to reduce hypoxia and modulate related signaling pathways. Hyperbaric oxygen therapy has been shown to reduce biomarkers of renal injury in diabetic patients (47). Additionally, modulating HIF signaling pathways may help alleviate hypoxia-related damage in DKD. Prolyl hydroxylase inhibitors (PHIs) are a class of drugs that act by stabilizing hypoxia-inducible factors (HIFs), particularly the HIF-α subunit. Clinical trials have demonstrated that PHIs can enhance HIF activity, offering the potential to alleviate diabetes-induced kidney hypoxia (48). Although these interventions have shown promising in preclinical and clinical studies, further research is needed to increase the efficacy and safety of DKD treatment.

### 3.4 Inflammation

Inflammation plays a crucial role in the pathophysiology of DKD. Systemic inflammation is common in patients with type 2 diabetes. A large number of pro-inflammatory factors such as interleukins IL-6, IL-8, IL-1β, monocyte chemoattractant protein-1(MCP-1), ROS and T cell activation secretion regulator can infiltrate into the renal capsule from an abnormal glomerulus and destroy the function of the renal tubules (49). A study observed that the STZ-induced diabetic rat showed upregulated macrophage inflammatory protein 3α (MIP-3α) expression in the renal cortex without prominent structural or functional manifestations of DKD during the first three weeks of hyperglycemia. Furthermore, MIP-3α expression is mainly distributed in the renal tubules and is responsible for activated macrophage infiltration (50). Thus, inflammatory processes are not only a consequence of renal injury, but are also involved in the pathogenesis of DKD (50).

Hyperglycemia, AGEs, mitochondrial oxidative stress and ultrafiltration cytokines act on renal tubular cells and trigger multiple cellular pathways such as PKC-α, -β, and -δ, NF-κB, P38 and ERK1/2 pathways, further exacerbating renal tubular cell hypertrophy, apoptosis, and accumulation of tubulointerstitial inflammatory factors (3). Accumulation of T cells and macrophages has been observed in animal models and patients with DKD (51). AGEs stimulate PTECs to upregulate the expression of IL-6 and intercellular adhesion molecule 1 (ICAM-1), which induce the transmigration of macrophages, lymphocytes and mast cells into the interstitial space (52). In addition, a large number of infiltrating inflammatory cells infiltrate and secrete abundant oxygen-free radicals and pro-inflammatory cytokines, which further induce renal damage and accelerate renal fibrogenesis and kidney injury (53). The accumulation of M2 macrophage cells was found to be closely associated with tubular atrophy and interstitial fibrosis (54). Toll-like receptors (TLRs) belong to the IL-1 receptor/toll-like receptor superfamily. TLRs are responsible for the recruitment of various adapter molecules and the initiation of both Myd88-dependent and independent pathways, leading to the engagement of NF-κB and interferon regulatory factor 3, as well as the production of downstream pro-inflammatory cytokines and chemokines. A number of studies have indicated that tubular TLR2 and TLR4 are the primary mediators of DKD (55, 56). Upregulation of TLR4 was observed in the renal tubules of DKD biopsies and positively correlated with interstitial macrophage infiltration. An *in vitro* study has shown that high glucose induces the expression of TLR4 in PTECs through the activation of PKC, resulting in the upregulation of CCL-2 and IL-6 expression. Along with the upregulation of TLR2 and TLR4, HMGB1 release and NF-κB activation were observed with increased cytokine expression in PTECs upon exposure to high glucose for 72h. In addition, albuminuria may also promote renal tubular inflammation in patients with DKD. A recent study has revealed that albuminuria can activate the Wnt/β-catenin signaling pathway in renal TECs, promoting the expression of TLR4/NLRP3-associated chemokines and inflammatory factors, thereby causing renal tubular and tubulointerstitial inflammation (57). Microarray data analysis has revealed the important role of inflammation-related signaling pathways in the pathogenesis of both glomerular and tubular DKD (58). The TECs of patients with DKD are affected by the inflammatory response, resulting in excessive production of intracellular inflammatory mediators which further activate apoptosis, glycolysis, and apoptosis-related pathways and induce renal tubular injury.

In summary, when the body is subjected to various injuries and stimuli, the inflammatory response is activated, and cytokines released by inflammatory cells can cause apoptosis, cellular dysfunction, oxidative stress, and increased expression of cell adhesion molecules in renal tubular epithelial cells, leading to structural and functional damage to the renal tubules. Although there is considerable evidence to suggest that inflammation is important in the progression of DKD, there is still a lack of large clinical trials to investigate the benefits of anti-inflammatory therapy in DKD (53, 59). Further clinical trials are needed to investigate the potential renoprotective effect of anti-inflammatory therapy.

### 3.5 Renin-angiotensin-aldosterone system

In DKD, the activation of renin-angiotensin-aldosterone system (RAAS) is commonly observed and contributes to the progression of DKD. RAAS plays a vital role in ensuring stable hemodynamics by controlling the volume of extracellular fluid, maintaining sodium balance, optimizing tissue perfusion, and exerting trophic effects on cardiovascular system. In DKD, continuous activation of RAAS leads to elevated pressure within the glomeruli, enhances the production of ROS, promotes tissue damage, triggers the release of proinflammatory cytokines, and stimulates the development of fibrosis in the glomeruli and tubulointerstitium.

Angiotensin II is the main effector of RAAS, which is a potent vasoconstrictor, abundantly expressed in the renal tubules. In DKD, upregulation of local angiotensin II in the renal tubule, activation of vascular endothelial growth factor (VEGF), and reduction of nitric oxide (NO) (60) can lead to constriction of both the afferent and efferent arterioles, as well as the interlobular arteries, compromising blood flow regulation. Furthermore, the expansion of the tubule-interstitial compartment contributes to the loss of capillaries, exacerbating the ischemic damage to proximal tubular epithelial cells (PTECs). In addition, elevated blood pressure may induce mechanical stress in the peritubular capillary network and mechanical shear stress. All the changes in the vascular factors mentioned above can lower post-glomerular blood flow during diabetes and contribute to further reductions in oxygen delivery to the tubules and hypoxia. Angiotensin II triggers the production of cytokines, such as TGF-β and MCP-1, and inflammatory and fibrotic factors, which contribute to the inflammatory response in the renal tubules and tubulointerstitial fibrosis (61–63). Angiotensin II enhances the reabsorption of sodium and water in the renal tubules by modulating the function of sodium transporters, specifically the sodium-hydrogen exchanger 3 (NHE3) and sodium-glucose cotransporter 2 (SGLT2). This mechanism leads to an increase in fluid volume and high blood pressure, which in turn causes additional damage to the tubular cells (64, 65).

Activation of RAAS also leads to the release of aldosterone, a mineralocorticoid hormone, from the adrenal glands. Aldosterone acts on the mineralocorticoid receptors (MR) in the renal tubules, promoting sodium reabsorption and potassium excretion. Excessive activation of MR leads to increased expression of pro-inflammatory and profibrotic genes, and generation of reactive oxygen species, contributory to tubular inflammation and fibrosis (66–68).

Overall, the activation of RAAS in DKD results in renal tubular injury through inflammation, hypoxia, fibrosis, and altered sodium and water handling. Targeting the components of RAAS, such as angiotensin-converting enzyme inhibitors (ACE inhibitors) or angiotensin receptor blockers (ARBs), is a common therapeutic approach to mitigate renal tubular injury and retard DKD progression. Recently, MR antagonists (MRAs) have been shown effective in alleviating DKD progression. The specific mechanism will be discussed in the following sections.

### 3.6 Epithelial–mesenchymal transition

EMT is a complex biological process in which renal tubular epithelial cells lose their characteristic features and acquire mesenchymal-like properties (69). This transition is accompanied by changes in cell morphology, gene expression, and cellular behaviour. EMT is characterized by the downregulation of cell-cell adhesion molecules, including E-cadherin, and the upregulation of mesenchymal markers, such as αSMA, fibroblast-specific protein 1 (FSP1), fibronectin, collagen, and vimentin (70).

In DKD, in response to severe or persistent inflammation, TECs undergo EMT through various signaling pathways, such as TGF-β, Wnt/β-catenin, PI3K/Akt, JAK/STAT, and Notch pathways (71–75). Meanwhile, fibroblasts and pericytes are activated. When intrinsic renal cells become activated, they secrete cytokines like TGF-β and CTGF that facilitate the formation of myofibroblasts which contribute to fibrosis by producing collagen I, collagen III, collagen IV, fibronectin, and laminin. This leads to the accumulation of extracellular matrix (ECM) and, ultimately, the development of tubulointerstitial fibrosis (76). As previously described, the RAAS becomes activated during DKD. Previous findings have shown that aldosterone triggers EMT by activating MR-mediated ERK1/2 pathway through mitochondrial-derived ROS in renal tubular epithelial cells (77).

Nevertheless, the precise detection of EMT in renal tissues remains challenging, and the development of effective anti-EMT therapies has been impeded for a long time. Comprehending the molecular mechanisms that drive EMT in DKD is vital to formulate precise therapeutic approaches against tubular injury. Further investigation is warranted to identify precise molecular targets and signaling pathways implicated in EMT.

### 3.7 Endoplasmic reticulum stress

ERS refers to the disruption of normal functioning and homeostasis within the endoplasmic reticulum (ER) of cells, resulting from the accumulation of unfolded or misfolded proteins and an imbalance between protein folding capacity and protein load. In DKD, ERS is primarily triggered by hyperglycemia, proteinuria, and the presence of AGEs and free fatty acids (78). The presence of these factors leads to the activation of pathogenic ERS and impairs the proper functioning of the unfolded protein response (UPR) pathway. G-protein coupled receptor 78 and phospho-eIF2α are markers of UPR and ERS. The overwhelming ERS results in tubular cell injury and ultimately contributes to the progression of kidney damage (79).

A recent study has revealed that ERS can increase the levels of unsaturated triacylglycerol precursors and reduce lipid droplet formation, leading to tubular injury and fibrosis in renal proximal tubular cell lines and STZ-induced diabetic mouse kidneys (80). In human proximal tubular epithelial cell line, ERS could induce apoptosis, EMT and ECM accumulation (81, 82). The expression of reticulon-1A (RTN1A), an ER-associated protein, was found to be elevated in diabetic kidneys of both humans and mice, and the expression of RTN1A is associated with the progression of DKD (83). Specific overexpression of RTN1A in TECs exacerbated DKD in mice through regulation of RTN1A-mediated ER-mitochondrial contact, as evidenced by increased tubular inflammation and apoptosis, tubulointerstitial fibrosis, and deterioration of kidney function (83). In addition, the activated UPR and hence the increased ERS further exacerbates inflammation and hinders protective processes like mTORc activation and autophagy (84).

Several studies have demonstrated the beneficial effects of various inhibitors targeting ERS in DKD. Sitagliptin, a dipeptidyl peptidase-4 inhibitor, ameliorate ERS in both albumin-treated PETCs and the kidneys of diabetic DBA2/J (D2) mice through upregulation of SIRT1 (85). A deeper understanding of the role and mechanisms of action of ERS in DKD tubular injury would contribute to the development of novel therapeutic targets.

### 3.8 Programmed cell death

Programmed cell death (PCD) is a naturally occurring process of cellular self-destruction that takes place in multicellular organisms. There are several different types of PCD, classified based on the mechanisms involved and stimuli that trigger the process. These include apoptosis, autophagy, pyroptosis, and ferroptosis. Recent research has shown that PCD is involved in the renal tubular injury observed in DKD.

#### 3.8.1 Apoptosis

Apoptosis is a natural and programmed form of cell death characterized by cellular shrinkage, chromatin condensation, and DNA fragmentation (86). Upon exposure to certain stimuli, cells acquire the apoptotic signal and apoptotic regulatory molecules interact sequentially to initiate the PCD pathway (87). Accumulating evidence has confirmed the critical involvement of TECs apoptosis in the pathophysiology of DKD. In both human and experimental studies, apoptotic cells have been found in the tubular epithelium of diabetic kidneys (88). Increased production of ROS increases oxidative stress and therefore promotes apoptosis via multiple caspase pathways in PTECs upon exposure to high glucose (89, 90).

Apoptosis is also closely associated with mitochondrial damage. In a cross-sectional study, mitochondrial fragmentation was specifically observed in the proximal tubules of kidneys from DKD patients. Damaged mtDNA accumulation and fragmented mitochondria lead to increased production of ROS and activate apoptosis in the tubules (91). In addition, a number of studies have identified possible molecules and signaling pathways closely related to TEC apoptosis, including protein arginine methyltranferase-1 (92), advanced oxidation protein products (93), and Bcl-2 interacting mediator (94). However, the comprehensive mechanisms remain to be elucidated.

In diabetic patients, hyperglycemia and other metabolic abnormalities cause oxidative stress and mitochondrial damage in renal TECs, thereby activating apoptotic pathways. Apoptosis of renal TECs leads to structural and functional damage to the renal tubules. Therefore, reducing renal tubular cell apoptosis is an important treatment goal for DKD. There have been studies exploring drugs and interventions that can potentially reduce renal tubular apoptosis in various kidney diseases, particularly DKD. For example, as a vasoprotective compound, calcium dobesilate is used for the treatment of diabetic retinopathy and chronic venous insufficiency. Previous findings have shown that calcium dobesilate could decrease the expression of BIM and inhibit apoptosis in high-glucose-treated proximal renal tubular cells (95). Also, a recent study has demonstrated that prostaglandin E1 could attenuate high glucose-induced apoptosis in proximal renal tubular cells by inhibiting the JNK/Bim pathway in streptozotocin-induced diabetic rats (96). Further research, including clinical trials, is needed to establish the effectiveness of these drugs in reducing renal tubular apoptosis and their effect on overall kidney function and disease progression.

#### 3.8.2 Autophagy

Autophagy is the key to maintain the stability of the intracellular environment, and is also a stress-responsive mechanism in pathophysiological conditions. Autophagy dysfunction can lead to undesirable cell damage or and apoptosis (97). Autophagy dysfunction has been demonstrated in renal tubular cells in both diabetic kidneys and experimental models of DKD (98). Renal tubules, especially PTECs often maintain high levels of autophagy to maintain their structural and functional integrity (99, 100). Dysregulation of autophagy in PTECs could lead to morphological alterations including hypertrophy, hyperplasia and EMT which further lead to renal structural changes such as renal hypertrophy, tissue injury and interstitial fibrosis (101, 102). STZ-induced diabetic rats had significantly fewer autophagic vacuoles and an accumulation of autophagy substrates (103). High glucose may contribute to impaired autophagy and increased senescence in PTECs, resulting in the accumulation of unfolded proteins and defective organelles (101). TGF-β is a critical mediator in regulating tubular cell death and renal fibrosis under diabetic conditions. As a result, it has been confirmed that inhibiting the removal of damaged cell contents may cause renal cell damage and fibrosis through TGF-β signaling pathway (104). AMPK, mammalian target of rapamycin (mTOR), cAMP-dependent protein kinase A (PKA) signaling, c-Jun N-terminal kinase 2 (JNK2) and TLR4 signaling have been implicated in autophagy regulation (87). Hyperglycemia can inhibit autophagy activity in proximal tubular cells by activating mTOR Complex 1 (mTORC1), a nutrient status sensor involved in cell growth, development and proliferation (105). Excessive albumin uptake and degradation in PTECs suppresses autophagy through an mTOR-mediated mechanism (106), resulting in tubular injury in DKD. Regulation and achievement of autophagy is controlled by autophagy-related genes (ATGs) (107). Alteration of ATG genes have been found in diabetic mouse models, including the deletion of Atg5 and Atg7 in PTECs, which is associated with the accumulation of damaged mitochondria, tubular cell apoptosis and renal fibrosis (108). Klotho is a transmembrane protein that acts as a coreceptor for fibroblast growth factor 23 (FGF23), a hormone involved in phosphate and calcium homeostasis. Klotho plays a crucial role in modulating FGF23 signaling and its effects on target tissues, primarily the kidneys (109). Klotho was first identified as an anti-aging factor that is mainly expressed in renal tubular cells (110). *In vivo* and *in vitro* studies have shown that Klotho enhances renal tubular cell autophagy and contributes to kidney protection via activation of AMPK and ERK and contributes to kidney protection pathways. However, the expression of Klotho and autophagic activity were remarkably suppressed in DKD mice and renal proximal tubule cells exposed to high glucose (111). Several preclinical studies have demonstrated that exposure to vitamin D can modulate the activity of the Klotho protein. In a previous study, researchers treated mice with a vitamin D-enriched diet and observed an increase in Klotho expression (112). In addition, Vitamin D treatment resulted in a significant upregulation of Klotho mRNA expression in various mouse renal cell lines (113), as well as in human proximal kidney cells (114). Besides, a phosphate-restricted diet upregulated the expression of Klotho in the kidneys of a mouse model of polycystic kidney disease (115). These preliminary findings urge future in-depth studies on how to modulate Klotho expression through dietary interventions to improve DKD.

Autophagy dysregulation is involved in the pathophysiology of DKD by increasing ROS production, mediating inflammatory responses and inducing renal cell damage and apoptosis (116, 117). Some studies have explored the use of drug intervention and dietary regulation to activate autophagy and protect the health of renal tubular cells. For example, some studies have found that mTOR inhibitors, such as rapamycin, sirolimus, and everolimus, can restore autophagic function and reduce renal damage (118). In addition, some dietary interventions, such as restricting calorie and protein intake, have been shown to activate autophagy and protect renal tubular cells (101). Although the impairment of autophagy in DKD is widely accepted, the regulation of autophagy and its underlying mechanism are still not fully understood. More extensive studies are needed to investigate the underlying detailed mechanism of autophagy in DKD.

#### 3.8.3 Pyroptosis

Pyroptosis is a novel type of PCD, distinct from apoptosis and autophagy. In DKD, high glucose levels and other metabolic abnormalities activate inflammasomes in TECs, which then cleave the proinflammatory cytokine pro-IL-1β into its mature form, IL-1β. IL-1β then activates pyroptosis by binding to its receptor on the cell membrane, resulting in the formation of gasdermin D (GSDMD) pores that cause cell membrane rupture and the release of proinflammatory cytokines and damage-associated molecular patterns (DAMPs), such as high-mobility group box 1, heat shock proteins, and fibronectin advanced glycation end-products IL-33. This triggers an inflammatory response that can exacerbate tubular injury and promote the progression of DKD (119). Recent research has shown that hyperglycemia induced cellular stress, such as ERS (120) and oxidative stress (119), which stimulate the pyroptotic process in renal cells, and various signaling pathways, such as TLRs/NF-κB/NLRP3 inflammasome signalling pathway, TXNIP/NLRP3 inflammasome pathway, ATP/P2X purinergic signalling pathway, and MAPKs/NLRP3 inflammasome signalling pathway (119, 121). Furthermore, the progression of DKD is strongly associated with pyroptosis-induced inflammation and cellular damage, which exacerbate renal fibrosis, glomerular sclerosis and tubular injury (122). IL-1β and IL-18, two crucial pro-inflammatory cytokines synthesized during pyroptosis regulation, are implicated in the pathogenesis of inflammatory responses in renal resident cells due to their association with the expression of chemoattractant cytokines and adhesion molecules (119). Researchers identified the upregulated expressions of active caspase-1, active N-terminal fragments of GSDMD (GSDMD-NT), IL-18 and IL-1β in the kidneys of db/db mice (119). Moreover, in db/db mice and diabetic patients, the tubular injury and tubular epithelial cell pyroptosis are accompanied by upregulated expression of NLRP3 inflammasome, IL-1β and TGF-β (123). Researchers have found high glucose can activate inflammasome, cause inflammatory cytokines release, and induce pyroptosis in proximal tubule epithelial cell line (124).

Pyroptosis can be identified by a combination of specific markers, which include TLR4, GSDMD, activation and release of pro-inflammatory IL-1β and IL-18, as well as the activation of cysteinyl aspartate specific proteinases, such as caspase-1, caspase-3, caspase-4, caspase-5, and caspase-11 (125). Researchers have found that human proximal tubule epithelial cell lines exhibited distinct ultrastructural alterations indicative of pyroptosis when exposed to high glucose, and that the expression of TLR4, NLRP3, caspase-1, GSDMD-N, IL-1β, and IL-18 are significantly upregulated (124). Besides, TECs from patients with DKD exhibited a significant increase in immunostaining levels of TLR4, GSDMD, NLRP3, IL-1β, and IL-18, which are common markers for pyroptpsis (124, 126, 127). Expressions of cleaved Caspase-1, GSDMD-NT, IL-18, and the secretion of IL-1β are also increased in the kidneys of db/db mice (127). However, intraperitoneal injection of TAK-242, an inhibitor of TLR4, protects tubular cell injury by suppressing GSDMD-related pyroptosis in db/db mice, and the expression of caspase-1 and GSDMD-NT were significantly decreased in renal cortex tissue (127).

In conclusion, pyroptosis in tubular cells plays an important role in the progression of DKD, and the underlying detailed mechanism requires further investigation. Inhibition of pyroptosis may be a promising therapeutic strategy for DKD.

#### 3.8.4 Ferroptosis

Ferroptosis refers to a specific PCD paradigm resulting from unrestrained lipid peroxidation (128). Ferroptosis is fueled by iron-dependent phospholipid peroxidation and is regulated by various cellular metabolic pathways, including iron handling, redox homeostasis, mitochondrial activity, amino acid, lipid and sugar metabolism, and various disease-related signaling pathways (129). The primary mechanism of ferroptosis is the catalysis of highly expressed unsaturated fatty acids on the cell membrane by divalent iron or esteroxylase, resulting in lipid peroxidation and cell death. It is also characterized by iron overload and downregulated expression of anti-oxidant proteins, including glutathione peroxidases (GSH-Px), superoxide dismutase (SOD), catalase (CAT) and glutathione peroxidase 4 (GPX4).

Ferroptosis frequently occurs in the tubules during the progression of renal disease due to the vulnerability of renal tubules to oxidative stress and lipid peroxidation (130). As a key mediator of ferroptosis, GPX4 is mainly expressed by TECs and is downregulated in DKD patients (131). Furthermore, GPX4 deficient mice are associated with increased interstitial oedema and tubular cell death, suggesting a critical role for GPX4 in mediating renal tubular injury (132). Polyunsaturated fatty acid (PUFA) accumulation in renal tubular cells renders the kidney susceptible to ferroptosis induced by lipid hydroperoxides (133). Furthermore, ferroptosis has been shown to exacerbate renal tubular injury via the HIF-1α/ Heme Oxygenase-1 (HO-1) pathway in diabetic mice (134). However, the role of ferroptosis in the progression of DKD remains controversial. A recent study found that drug-induced ferroptosis contributes to the clearance of senescent tubular cells, with potential benefits for kidney disease (135). Further studies should be conducted to elucidate the detailed role of ferroptosis in renal tubular cells during the progression of DKD.

Autophagy, apoptosis, pyroptosis and ferroptosis are all parts of regulated cell death and are somehow linked. Autophagy promotes ferroptosis by degrading ferritin. On the other hand, erastin-induced ferroptosis is suppressed by downregulating Atg5 and Atg7 through reducing intracellular ferrous iron levels and lipid peroxidation (136). Apoptosis of TECs is suppressed by autophagy activation, while autophagy inhibition is associated with apoptosis activation (87). An interaction between apoptosis and pyroptosis also exists in the renal tubules of DKD patients. In DKD, overexpression of GSDMD induces pyroptosis and reduces apoptosis, thereby switching from apoptosis to pyroptosis in TLR4-mediated renal TECs injury (137). PCD of TECs is essential for hyperglycemia-mediated tubular injury in DKD. Further studies should be performed to clarify the interactive mechanisms between different types of cell death in tubular injury of DKD.

## 4 Treatment

DKD is a chronic complication of diabetes, improvements in glycemia control and blood pressure management by blocking the RAAS, currently available therapies could not be able to prevent progression to ESRD completely. Previously, we have described the mechanism underlying the development tubular injury of DKD, here we reviewed the protective effect of hypoglycaemic drugs on renal tubules (**Table 1**), and some new therapeutic strategies, such as stem cell therapies.

**Table 1:** Hypoglycemic agents mentioned with renal-protective effect.

Hypoglycemic agents	Renoprotective mechanisms	Reference
**Metformin**	a. ameliorate inflammation b. suppress oxidative stress c. regulate apoptosis, autophagy d. regulate cellular senescence	a (138–141). b (142). c (141, 143–145). d (146).
**SGLT-2i**	a.improve tubular function: improve glomerular haemodynamics, reduce eGFR and albuminuria b. suppress inflammatory, c.anti-oxidative stress d. regulate energy metabolism e. improve autophagy	a (147–151). b (152–159). c (160, 161). d (155, 162, 163). e (164–168).
**DPP-4i**	a.anti-inflammatory b. anti-apoptosis c. antioxidant response	a (169–172). b (173). c (174).
**GLP-1/GLP-1RA**	a. reduce proteinuria, eGFR and hyperfiltration b. anti-inflammatory c. modulate the RAAS system	a (175, 176). b (175). c (177, 178).

### 4.1 Metformin

Metformin has been widely used in the treatment of T2DM. In addition to its benefits on glycemia, its reno-protective effect has been recognized gradually. Metformin has been shown to be associated with a decreased death risk, improved clinical outcomes, and delayed progression to ESRD in patients with DKD (179). Experiments both *in vitro* and vivo have shown that metformin attenuates the progression of DKD by suppressing renal inflammation, oxidative stress, and fibrosis (180). In diabetic mice, metformin treatment has been shown to regulate autophagy, downregulate apoptosis, partially restore renal function and increase survival. The reno-protective effect of metformin may be partly due to downregulated inflammation (138–140). Metformin exerts its anti-inflammatory effects through the AMPK signaling pathway. Metformin also inhibits NLRP3 inflammasome (141). Metformin could suppress the accumulation of hypoxia-induced HIF-1α and expression of the target genes in human renal TECs (142). In a renal proximal tubule-specific Tsc1 gene-knockout mouse model, downregulated AMPK phosphorylation was observed in the enlarged kidneys, which also exhibited aberrant proliferation of proximal tubule cells, renal interstitial inflammation, and fibrosis. Metformin treatment significantly increased AMPK phosphorylation while decreased the Akt phosphorylation, resulted in inhibition of proliferation and induction of apoptosis in the renal proximal tubule cells (143). Metformin also inhibits the development of DKD by inducing autophagy. Under hyperglycemia, AMPK phosphorylation is inhibited while EMT is activated in renal TECs. Metformin-induced AMPK activation significantly improved renal autophagy, inhibited EMT of renal TECs, reduced tubulointerstitial fibrosis and delayed the onset of DKD (144). Metformin has been demonstrated to prevent renal tubulointerstitial fibrosis (181) by addressing the reduced energy supply resulting from impaired fatty acid oxidation (FAO) in proximal tubular cells. In high fat diet (HFD) and streptozotocin (STZ)-induced type 2 diabetic mice model and HK-2 cells, after treatment with metformin (200 mg/kg/d) for 24 weeks, some indicators of mitophagy (LC3II, Pink1, Parkin, Atg5) were examined. The expression of p-AMPK, Pink1, Parkin, LC3II, and Atg5 in renal tissue of diabetic mice were remarkably decreased. In addition, metformin activated p-AMPK and promoted the translocation of Pink1 from the cytoplasm to mitochondria, thereby promoting the occurrence of mitophagy in HK-2 cells under HG/HFA ambience (141). Metformin could improve the mitochondrial mass of renal TECs *in vitro* upon high glucose exposure (141). Metformin improves diabetic tubulopathy by modulating mitochondrial dynamics and autophagy. Treatment with metformin improved functional mitochondrial mass in HKC8 cells in high-glucose conditions and it is correlated with the reversal of changes in Drp1, Mfn1, and LC3-II protein expression. Normalized mitochondrial life cycles resulted in low ROS production and reduced apoptosis. Metformin treatment mitigated albuminuria and renal histopathology, and decreased the expression of TGFβ1 and αSMA in the kidneys of STZ-induced C57/BL6J diabetic mice (145). More importantly, metformin treatment was shown to suppress senescence of mouse renal TECs under hyperglycemic condition (182).

In conclusion, metformin could ameliorate inflammation and oxidative stress in renal tubular cells, regulate apoptosis and autophagy, and attenuate tubulointerstitial damage through various mechanisms. Existing experimental and clinical evidence suggests promising effects of metformin against DKD.

### 4.2 SGLT2 inhibitors

SGLT2 expresses in S1 and S2 segments of the proximal tubules of kidney. It plays an important role in the reabsorption of glucose from urine. In the diabetic kidney, the expression levels of SGLT2 were shown to be upregulated significantly (183). According to this essential biological function, the inhibitors of SGLT2 (SGLT2i, such as dapagliflozin, empagliflozin, ipragliflozin and tofogliflozin) have been developed to release more glucose from urine by blocking glucose reabsorption mediated by the proximal tubules (184). Because of this property, their beneficial effects on DKD have been discovered gradually.

SGLT2i has multiple benefits, such as delaying the progression of kidney disease and improving renal filtration (147). It also suppresses the processes associated with DKD, such as albuminuria and increased kidney weight, to improve tubular function significantly (148). The main mechanism of SGLT-2i is to act on the SGLT2 receptor of the proximal tubule, inhibit the reuptake of Na^+^ and glucose, increase the excretion of glucose in urine to reduce blood glucose, and subsequently increase natriuria. This results in an increase of Na^+^ at the macula densa, TGF activation, reduced glomerular hyperperfusion, high pressure, high filtration, and the recovery of renal function (149). In the KK/TA-Ins2Akita mice treated with a combination of empagliflozin and rigagliptin, which provided greater reductions in glomerular albumin filtration and GFR, as well as higher urinary sodium excretion, the dual inhibition of SGLT2 and DPP-4 highly promoted distal tubule sodium delivery, compared with empagliflozin monotherapy. Dual inhibition of SGLT2 and DPP-4 highly promotes a distal tubular sodium delivery and thereby contributes to the appropriate modulation of preglomerular arteriolar tone and intraglomerular pressure via an increase in adenosine release and a reduction in PGE2 secretion from macula densa during DKD (150). In T2DM patients, SGLT2i treatment significantly reduced the risk of progression to DKD by 45% (151). In clinical trials, several SGLT2i agents have been shown to reduce eGFR and albuminuria significantly (147).

SGLT2i inhibits diabetic kidney growth due to the glucose-lowering effect. In empagliflozin-treated mice, renal growth was significantly attenuated, and molecular markers of renal growth were reduced in proportion to the reduction in hyperglycemia. In a type 1 diabetic Akita mouse model, which was used as a nonobese insulin-dependent model of spontaneous type 1 diabetes, empagliflozin treatment attenuated diabetes-induced rise in renal expression of markers of kidney growth by reducing the cyclin-dependent kinase inhibitors p27 and p21, which were over-expressed in early diabetic kidneys (152). In population studies, cagaglizin, dagaglizin, and enaglizin slightly reduced serum markers of inflammation, such as hsCRP, TNF-a, IL-6, and IFN-γ (153). In a db/db mouse model of type 2 diabetes, dapagliflozin treatment for 12 weeks inhibited the expression of TGF-β , MCP-1, osteopontin and ICAM-1, and thereby inhibited diabetes-induced inflammation in the kidneys (154). It could limit and prevent the overproduction of ROS (160) and suppress the activation of the NLRP3 inflammasome in the renal tubules (155). SGLT2i treatment significantly suppresses the expression of inflammatory makers in renal tubules, such as IL-1β, IL-6, TNF-α, and tumor necrosis factor receptor-1 (TNFR-1) (156). The anti-inflammatory mechanism of SGLT2i is also related to the HIF signaling pathway, activated AMPK signaling pathway and mitochondrial energy metabolism (162). SGLT2i could inhibit aberrant glycolysis (163). Restoration of SIRT3 levels by SGLT2i is central to the regulation of antiglycolytic systems and activation of antioxidant and antifibrotic signaling cascades. In the ischemia/reperfusion-induced fibrosis C57BL/6J mouse model, transcriptomic and metabolomic analyses showed that dapagliflozin treatment mitigated accumulation of tricarboxylic acid (TCA) circulating metabolites and up-regulation of inflammation in fibrotic renal cortical tissue. Dapagliflozin relieved the activation of mTOR and HIF-1α signaling, and restored tubular cell-preferred fatty acid oxidation in PTECs. The activation of NOD-, LRR-, and NLRP3 inflammasome were strikingly blocked by dapagliflozin. The immunomodulatory metabolite itaconate derived from the TCA cycle was significantly boosted as a result of decreased isocitrate dehydrogenase 2 and increased immune-responsive gene 1 and mitochondrial citrate carrier in dapagliflozin-treated mice, accounting for the inhibitory effect of dapagliflozin on NLRP3 inflammasome activation (155).. Oxidative stress and DNA damage were rescued in the glomerulus of db/db mice treated with dapagliflozin. In H_2_O_2_-treated HK-2 cells (human renal tubular epithelial cell line), dapagliflozin increased plasma β-HB, induced NRF2 nuclear translocation and activated downstream antioxidant pathways (161).. Meanwhile, in cultured proximal tubular cells from human normal kidney, tofogliflozin was shown to suppress high glucose-induced ROS generation, MCP-1 induction and apoptosis. Blockade of glucose reabsorption in tubular cells by tofogliflozin could exert beneficial effects on tubulointerstitial damage in diabetic nephropathy by directly preventing the glucotoxicity to proximal tubular cells. Given the pathological role of oxidative stress generation in MCP-1 induction and apoptotic cell death in tubular cells, hyperglycemia-elicited ROS generation might be a therapeutic target of tofogliflozin (157, 158). AMPK phosphorylation was suppressed, and autophagy activity was inhibited in high-glucose cultured human renal proximal epithelial cells (HRPTCs) and STZ-induced diabetic nephropathy mice. After the intervention of SGLT-2 inhibitor empagliflozin, AMPK activity was restored, LC3II expression was enhanced, p62 protein expression was decreased, and autophagy activity was restored (164). Similarly, in STZ-induced diabetic rats, activation of SIRT1 activity can positively regulate autophagy (165). Previous study has shown that SGLT-2 inhibitors can up-regulate SIRT1 in renal tubules (166), thereby positively regulating autophagy and improving proteinuria (167). Activated AMPK inhibits mTORC1 activity and enhances autophagy (168).

### 4.3 Dipeptidyl peptidase-4 enzyme inhibitors

Glucagon-like peptide-1 (GLP-1) is a peptide hormone secreted by intestinal cells that stimulates insulin secretion from pancreatic β cells in response to blood glucose. GLP-1 could be rapidly degraded by dipeptidyl peptidase-4 (DPP-4) (185). DPP-4 inhibitors (DPP-4i; sitagliptin, vildagliptin, alogliptin, et al) are classical anti-hyperglycemic agents that suppress GLP-1 degradation and prolong the action of the glucose-dependent insulinotropic polypeptide. DPP-4i also has reno-protective effects independent of glycemic control. Recent studies have shown that DPP-4i not only regulates glucose homeostasis but also plays a reno-protective role against DKD. Sitagliptin induces natriuresis by blocking distal tubular sodium reabsorptive mechanisms rather than proximal sodium uptake, which can be modulated by SGLT2 inhibitors. These renal natriuretic effects occur distal to the macula densa. In addition, DPP-4 modulates multiple substrates such as GLP-1, BNP, substance P, and neuropeptide Y (NPY), leading to potential extra-pancreatic effects, particularly the promotion of sodium excretion in urine (186). In DKD patients, gemigliptin treatment significantly suppressed the expression of renal tubular injury biomarkers and the levels of vascular calcification markers (187). DPP-4i also has an anti-inflammatory effect. DPP-4i could restore the inactivation of AKT and ERK signaling and inhibit NF-κB activation to reduce the production of pro-inflammatory factors, including TNF-α, IL-1β, monocyte chemoattractant protein-1 (MCP-1), ICAM-1 and TGF-β (169, 170). DPP-4i treatment also facilitated the reduction of NLRP3-mediated inflammation in a rat model of renal fibrosis (171).

DPP-4i could regulate the redox state in the kidney. Sitagliptin downregulated Keap-1 expression in diabetic kidney to activate the antioxidant response through the Nrf2 signaling pathway and improved renal morphology and clinical manifestations (174). DPP-4i could reduce renal tubular damage associated with DKD by improving mitochondrial homeostasis via the stromal cell-derived factor 1/C-X-C chemokine receptor type 4 pathway. DPP-4i treatment significantly increases proximal tubular cell viability and reduces ROS production.

DPP-4i plays an anti-apoptotic role by regulating the mitogen-activated protein kinase (MAPK)/ERK, phosphatidylinositol-3-kinase (PI3K)/AKT and NF-κB signaling pathways. Previous population studies have shown that AGEs significantly increased the expression of DPP-4 and promoted the release of soluble DPP-4 in renal tubular cells (173). In addition, DPP-4i may protect against albuminuria by targeting EMT in renal TECs resulting from the interaction between DPP-4, integrin β1 and caveolin-1 (188). Furthermore, DPP-4i could inhibit the expression of MCP-1 in mouse proximal TECs upon free fatty acid binding to albumin and significantly attenuate renal tubulointerstitial injury (172).

### 4.4 Glucagon-like peptide-1/GLP-1 receptor agonists

Endogenous and pharmacological activation of the GLP-1 receptor (GLP-1R) axis is thought to be reno-protective in DM (177). GLP-1R agonists (GLP-1RAs) are recognized as a new class of hypoglycemic agents with potential for the prevention and treatment of DKD (189). GLP-1 and GLP-1RA treatment have been shown to decrease proteinuria, eGFR and hyperfiltration (175). Exenatide-loaded microspheres were shown to reduce albuminuria by 26% in T2DM patients, independent of their glucose-lowering effect (176).

Recombinant human GLP-1 (rhGLP-1) could reduce urinary albumin without altering body weight or food intake. It reduced the pro-fibrotic factor collagen in renal tubular tissue and human proximal tubular cells by inhibiting the NF-κB and MAPK signaling pathway in DM. Meanwhile, rhGLP-1 treatment suppressed the expression of α-smooth muscle actin (α-SMA), fibronectin and inflammatory factors including MCP-1 and TNFα (175).

GLP-1 has a natriuretic effect in the proximal renal tubules, the beneficial effects of GLP-1 and GLP-1RA may be related to the regulation of the RAAS (177). GLP-1RA might exert reno-protective effects by stimulating proximal tubular natriuretic peptide in the proximal tubules, modulating cAMP/PKA signaling and inhibiting RAAS (178). Animal studies have reported the presence of GLP-1R mRNA in the glomerulus and the initial part of proximal convoluted tubes (190). In human kidneys, GLP-1R has been identified as localized to proximal renal tubule cells and preglomerular vascular smooth muscle cells (191). GLP-1 has been demonstrated to induce natriuresis and diuresis, likely involving the inhibition of the sodium–hydrogen exchanger 3 (NHE3) localized at the brush border of the renal proximal tubular cells (192). Liraglutide, an incretin GLP-1 mimetic, can increase atrial natriuretic peptide (ANP) secretion to promote urinary sodium excretion, via the mediation of NHE3 phosphorylation. In addition, GLP-1RA can also indirectly affect RAAS by reducing the circulating level and action of renin and AngII, playing a role in sodium excretion and diuresis by regulating the ion exchange of potassium, chlorine and calcium in renal tubules (178). Short-term treatment with liraglutide lowered ANG II concentrations by 21% and limited sodium reabsorption in the proximal renal tubules. It plays a reno-protective role by reducing Ang II levels (193). Treatment with GLP-1RA significantly attenuated high glucose-induced tubule damage in DKD kidney tissues and TECs cells (177).

These findings suggest that both DPP-4i and GLP-1RA elicit pleiotropic tubular protective effects and may serve as another potential therapeutic strategy options to benefit patients with DKD.

### 4.5 Anti-inflammation therapies

We have discussed the crucial role of inflammation in DKD tubular injury. Here we summarize the current research progress of anti-inflammatory therapies in the treatment of DKD. By targeting the inflammatory pathways involved in the disease, these therapies aim to reduce inflammation, oxidative stress, and the release of pro-inflammatory mediators in the renal tubules.

Previous study has found that RAS blockers can reduce proteinuria and tubulointerstitial fibrosis not only through their hemodynamic/antihypertensive effects but also through the inhibition of NF-κB, MCP1 gene expression, and macrophage infiltration, thereby exerting anti-inflammatory/antifibrotic effects in the STZ-induced diabetic rat model (194). Furthermore, in line with the beneficial effects of RAS blockers, researchers have also shown that the administration of empagliflozin, a SGLT2i, reduced the expression of IL-6, CCL2, and NF-κB in the kidneys of diabetic Akita mice, as well as in cultured kidney tubular cells exposed to high glucose (152, 159). TLR2 and TLR4 signaling have been shown to play a significant role in renal tubular inflammation in DKD. Empagliflozin was found to diminish the expression of TLR4 induced by high glucose in a human kidney proximal tubule cell line (159). Increasing evidence indicates that high mobility group box 1 (HMGB1), a nuclear protein released from dendritic cells, macrophages, and necrotic cells during inflammation, plays an important pathological role in DKD by activating TLR2 and TLR4 signaling (195). However, high glucose-induced NF-κB activation, oxidative stress, and inflammation can be ameliorated by a HMGB1 inhibitor, glycyrrhizin, in human renal proximal tubular cells (196). Inhibition of the NLRP inflammasome is a promising novel therapeutic strategy for many inflammatory diseases (197). In db/db mice, administration of M-920, a caspase inhibitor, reduced renal CASP1, IL-1β, IL-18, and NLRP3 inflammasome activation, leading to amelioration of albuminuria and renal ECM accumulation (198). Animal studies have provided evidence that sitagliptin and linagliptin exhibit the ability to attenuate the activity of NLRP3/inflammasome (198). However, further investigations are warranted to elucidate their specific effects on renal tubules during DKD. Furthermore, several inhibitors targeting inflammatory signaling pathways, such as Baricitinib, Sirukumab, and Emapticap pegol, have demonstrated efficacy in clinical trials for improving eGFR, UACR, and retarding the progression of kidney disease (199–201). However, the effectiveness and safety of these inhibitors require further confirmation through larger-scale clinical trials.

It is worth noting that non-specific anti-inflammatory treatment for DKD may potentially enhance susceptibility to infections (202). A deeper understanding of the underlying mechanisms could lead to the identification of more specific and less toxic therapeutic targets for the treatment of DKD.

### 4.6 Anti-senescence therapies

Cellular senescence in TECs is influenced by various factors, including hyperglycemia, oxidative stress, inflammation, accumulation of AGEs, lipotoxicity, and disturbances in lipid metabolism. It can be triggered by inhibiting AMPK–mTOR signaling, overexpressing Wnt9a activating p21 or the Wnt–β-catenin pathway (203). The extent of tubular senescence is associated with the severity of kidney injury, decline in renal function, and the development of renal fibrosis (204). Targeting tubular cell senescence in DKD may emerge as a crucial therapeutic target for the treatment of DKD.

Researchers have found that resveratrol and metformin provide protection to proximal tubular cells against high glucose-induced EMT and cellular senescence by activating the AMPK/mTOR signaling pathway (146). Klotho, an anti-aging protein, was shown to attenuate mitochondrial injury, cellular senescence, and renal fibrosis induced by Wnt1 and Wnt9a in both unilateral ischemia-reperfusion mice and cultured human renal tubular epithelial cells (205). The pathway of p21-dependent tubular senescence plays a significant role in the development of hyperglycemic memory in DKD (206). The protease activated protein C and parmodulin-2 were found to effectively counteract sustained tubular p21 expression and tubular senescence in both mouse proximal tubular cells and db/db mice (206). Recently, certain senolytic and senostatic agents, such as rapamycin, Nrf2 activators, and calorie restriction, have demonstrated effectiveness in ameliorating cellular senescence. However, further confirmation is still needed to determine their efficacy (207).

Although anti-senescence therapies show promising results in experimental models of DKD, their translation to clinical practice requires further investigation. Clinical trials are needed to assess the safety and efficacy of these approaches in humans and to determine their potential to halt or reverse the DKD progression.

### 4.7 Mineralocorticoid receptor antagonists

As we have discussed earlier, the activation of MR plays a crucial role in the pathogenesis of DKD. Novel medications that specifically target MR are being considered as promising therapeutic approaches to attenuate the progression of DKD and renal fibrosis. Finerenone, a novel non-steroidal selective MR antagonist (MRA), demonstrates fewer adverse effects on serum potassium levels and renal function compared to steroidal MRAs which have been approved by FDA for treatment of DKD (208). The results of large-scale clinical trial FIDELIO-DKD have revealed that finerenone can reduce kidney failure and DKD progression (209). In rats subjected to ischemia-reperfusion, the administration of finerenone effectively reduced tubular injury, tubulointerstitial fibrosis and suppressed the expression of TGF-β by mitigating oxidative stress (210). Administration of finerenone could downregulate the expression of the proinflammatory cytokines IL-6 and IL-1ß in the kidneys of bilateral ischemia-reperfusion-induced CKD in mice, and ameliorate inflammation and tubulointerstitial fibrosis. Finerenone exhibits greater potency in reducing fibrosis compared to eplerenone. It is suggested that the differential modulation of MR co-factors is associated with the specific ability of finerenone to improve the expression of tenascin X (TNX), an MR target gene that plays a crucial role in fibrosis regulation (211). In addition, Bhuiyan et al. observed that esaxerenone, a nonsteroidal selective compound, effectively decreased the expression of MCP-1 and inhibited the infiltration of inflammatory cells in DKD by suppressing ROS levels (212).

These findings suggest that MRAs elicit potential therapeutic effect in DKD. However, further clinical and basic research are needed to explore its underlying mechanisms.

### 4.8 Stem cell therapies

With the development of technology, stem cell-based therapy could be another therapeutic option for DKD. Common stem cell therapy options mainly target mesenchymal stem/stromal cells (MSCs), umbilical cord/amniotic fluid (UC/AF) cells, embryonic stem cells (ESCs), induced pluripotent stem cells (iPSCs) and stem cell-derived products. Tissue sources for stem cell therapy include bone marrow (BM), UC/AF, urine, and adipose tissue. Importantly, stem cells play a repairing role by migrating to the injured sites, inducing directional differentiation, eliciting paracrine effect and modulating the immune response. Cell-based therapy effectively improves renal function while reducing indicators of damage such as proteinuria, fibrosis, inflammation, apoptosis, EMT and oxidative stress (213). Here we summarize the common stem cell therapy strategies for the treatment of DKD.

#### 4.8.1 Treatment with MSCs

MSCs, perhaps the most extensively studied stem cells today, are a group of cells with differentiation and proliferative potential. MSCs could recognize the damaged tissue, followed by migration and integration into the specific sites (214). MSCs facilitate the repair of the injured kidney mainly through paracrine and immunomodulatory effects (214). MSCs can benefit damaged renal tubules by releasing a variety of trophic and immunomodulatory factors to trigger intracellular signaling in target or neighboring cells. These factors include VEGF, FGF, PDGF, IGF-1, HGF and EGF (215). MSCs could reduce the production of inflammatory factors such as IL, TGF, TNF and interferon (IFN), inhibit the proliferation of T and B cells, reduce IgG secretion, inhibit the maturation of dendritic cell and regulate the activities of other immune cells such as natural killer cells and macrophages (214). MSCs also modulate the pathogenesis of DKD by acting as anti-inflammatory mediators in renal TECs (216). MSCs can be transported to the kidney through arterial or intravenous injection, and directly act on the kidney through local injection or transplantation. Once MSCs are delivered to the kidney, they can recognize the damaged tissue, migrate to the specific sites of injury, and integrate into the renal microenvironment. The precise mechanisms underlying MSC homing and localization in the kidney are still being studied, but it is thought to be related to interactions with adhesion molecules, chemokines, and cytokines present in the injured tissue. MSCs improve renal tubular cell function by facilitating the M2 phenotype of macrophages and suppressing the adhesion and migration of M1 macrophages (217). Overall, MSCs induce a pro-regenerative microenvironment to delay the progression of DKD (218). By influencing SGLT2 expression, MSCs can potentially improve glycemia control and exert anti-inflammatory effects (219).

The main types of MSCs used in the treatment of DKD include adipose-derived MSCs (AD-MSCs), umbilical cord-derived MSCs (UC-MSCs), bone marrow-derived MSCs (BM-MSCs) and human urine-derived MSCs (HU-MSCs).

##### 4.8.1.1 AD-MSCs

AD-MSCs were isolated from adipose tissues of 7-week-old enhanced green fluorescent protein rats and AD-MSCs sheets were prepared. A DN rat model was established from 5-week-old SD Torii fatty rats. AD-MSCs were administered intravenously and transplanted directly into the kidneys. After AD-MSC transplantation, urinary albumin and urinary TNF-α levels were significantly decreased. Histologically, the structure of the renal tubular architecture was preserved (220).

##### 4.8.1.2 BM-MSCs

BM-MSCs could be obtained from the bone marrow through isolation of the mononuclear cell. Isolated monocytes containing BM-MSCs were cultured and expanded *in vitro* using specialized growth media. BM-MSCs can be transplanted using different methods depending on the target tissues or organs, such as intravenous injection, local injection and scaffold-based transplantation. BM-MSCs transplantation could benefit mitochondrial and induce mitochondrial transfer to rescue protect renal TECs, by inhibiting apoptosis and improved morphology, of tubular basement membrane and brush border structure. BM-MSC transplantation also enhanced the expression of mitochondrial superoxide dismutase 2 and Bcl-2, and inhibits the production of ROS. BM-MSCs increased the expression of megalin and SGLT2, and restore the structural restoration of renal tubules (221). Intravenous infusion of MSCs could effectively improve renal tubular function and prevent renal failure in diabetic mice by activating the PI3K/AKT signaling pathway (222). In addition, BM-MSCs could also promote the recovery of tubule damage (223).

##### 4.8.1.3 UC-MSCs

The major advantages of UC-MSCs include ease of collection, low immunogenicity and high paracrine potential (224). Animal studies have shown that UC-MSCs could migrate to the kidneys to effectively repair renal dysfunction by reducing proteinuria, serum creatinine and urea nitrogen levels and increasing creatinine clearance in DKD rats (225). UC-MSCs could also suppress the inflammatory response to ameliorate DKD-associated renal fibrosis (226).

##### 4.8.1.4 MSC-Exos

Extracellular vesicles with a diameter of 30 to 150 nm are known as MSC exosomes, which have the same lipid bilayer structure as the cell membrane (227). MSC exosomes could attenuate the damage induced by high glucose to podocytes (228). Animal studies have shown that MSC-Exos intervention could promote proliferation of renal tubular endothelial cells to protect renal function (215, 229). MSC-Exos can carry complex molecular cargoes such as proteins, lipids and nucleic acids, such as DNA, miRNA and circRNA. The contents of these cargoes serve a useful purpose. It was shown that stem cell exos protect the kidney from damage through multiple pathways by eliciting anti-apoptotic, anti-inflammatory, anti-oxidative, and anti-fibrotic effects and by regulating podocyte autophagy (230). Therefore, MSCExos are expected to be a potential therapeutic option for the treatment of DKD (231).

#### 4.8.2 Other stem cell therapies

iPSCs are pluripotent stem cells reprogrammed from terminally differentiated somatic cells, such as fibroblasts, keratinocytes, extraembryonic tissues, umbilical cord blood and peripheral blood cells. With a strong differentiation capacity, iPSCs could be induced to differentiate into renal tubular cells and podocytes (232). ESCs are multipotent cells derived from the cell mass within the blastocyst which can be induced to the cells of the kidney lineage. Both mouse and human ESCs can be induced to differentiate toward renal lineage by a series of defined growth factors or inducers (233, 234). By exposure to renal epithelial cell medium supplemented with a combination of bone morphogenetic protein, activin A and FGF, human ESCs were induced to differentiate into proximal tubular-like cells (234). ESCs differentiate through posterior primitive streak and intermediate mesoderm as normal nephrogenesis, then the ESC-derived kidney progenitors generate a self-organizing kidney after 3D culture (235). iPSCs and ESCs can enter damaged kidneys through systemic delivery via intravenous infusion (236), or local injection (237), as well as through scaffold-based approaches (238), providing potential for transplantation and regenerative therapies. Further works are needed to translate research on iPSCs and ESCs into actual therapies for DKD.

## 5 Conclusions

Tubular injury occurring in the early stages of DKD may have latent long-term consequences. In this review, we have discussed the potential mechanisms of renal tubular injury in DKD, including hyperglycemia, lipid accumulation, oxidative stress, hypoxia, RAAS, ER stress, inflammation, EMT and programmed cell death. Until recently, drugs that directly target renal tubular injury in patients with DKD have been limited. Hypoglycemic drugs such as metformin, SGLT2i, DPP-4i, GLP-1RA, anti-inflammation therapies, anti-senescence therapies, mineralocorticoid receptor antagonists, and stem cell therapies have also shown beneficial effects against tubular injury, potentially slowing the progression of the DKD, and thereby protecting the kidney. However, further studies are needed to elucidate the potential beneficial mechanisms of these drugs and therapies.

## Author contributions

Conceptualization, YW; Writing—original draft preparation, YW, and MJ; Writing—review and editing, CKC and QL; Supervision, CKC and QL; All authors have read and agreed to the published version of the manuscript.
